# Queueing Theory and COVID-19 Prevention: Model Proposal to Maximize Safety and Performance of Vaccination Sites

**DOI:** 10.3389/fpubh.2022.840677

**Published:** 2022-07-07

**Authors:** Marcello Di Pumpo, Andrea Ianni, Ginevra Azzurra Miccoli, Andrea Di Mattia, Raffaella Gualandi, Domenico Pascucci, Walter Ricciardi, Gianfranco Damiani, Lorenzo Sommella, Patrizia Laurenti

**Affiliations:** ^1^Department of Life Sciences and Public Health, Università Cattolica del Sacro Cuore, Rome, Italy; ^2^Hospital Management, Fondazione Policlinico Universitario Campus Bio-Medico, Rome, Italy; ^3^Hospital Pharmacy, Fondazione Policlinico Universitario Campus Bio-Medico, Rome, Italy; ^4^Department of Health Professions, Fondazione Policlinico Universitario Campus Bio-Medico, Rome, Italy; ^5^Department of Woman and Child Health and Public Health, Fondazione Policlinico Universitario Agostino Gemelli IRCCS, Rome, Italy

**Keywords:** COVID-19, vaccination site, queueing theory, physical distancing, safety and performance

## Abstract

**Introduction:**

COVID-19 (Coronavirus Disease 19) has rapidly spread all around the world. Vaccination represents one of the most promising counter-pandemic measures. There is still little specific evidence in literature on how to safely and effectively program access and flow through specific healthcare settings to avoid overcrowding in order to prevent SARS-CoV-2 transmission. Literature regarding appointment scheduling in healthcare is vast. Unpunctuality however, especially when targeting healthcare workers during working hours, is always possible. Therefore, when determining how many subjects to book, using a linear method assuming perfect adhesion to scheduled time could lead to organizational problems.

**Methods:**

This study proposes a “Queuing theory” based approach. A COVID-19 vaccination site targeting healthcare workers based in a teaching hospital in Rome was studied to determine real-life arrival rate variability. Three simulations using Queueing theory were performed.

**Results:**

Queueing theory application reduced subjects queueing over maximum safety requirements by 112 in a real-life based vaccination setting, by 483 in a double-sized setting and by 750 in a mass vaccination model compared with a linear approach. In the 3 settings, respectively, the percentage of station's time utilization was 98.6, 99.4 and 99.8%, while the average waiting time was 27.2, 33.84, and 33.84 min.

**Conclusions:**

Queueing theory has already been applied in healthcare. This study, in line with recent literature developments, proposes the adoption of a Queueing theory base approach to vaccination sites modeling, during the COVID-19 pandemic, as this tool enables to quantify ahead of time the outcome of organizational choices on both safety and performance of vaccination sites.

## Introduction

Since the report of a first suspected case on December 8, 2019 in Wuhan, Coronavirus disease 2019 (COVID-19) has rapidly spread all around the world (1). Induction of herd immunity by mass vaccination has been a very successful strategy for preventing the spread of many infectious diseases, hence protecting the most vulnerable population groups unable to develop immunity, for example individuals with immunodeficiencies or a weakened immune system due to underlying medical or debilitating conditions. Therefore, vaccination represents one of the most promising counter-pandemic measures to COVID-19 (2). Finding safe and effective vaccination models has, therefore, become a global priority.

However, there is still little specific evidence in literature on how to safely and effectively program access and flow through specific healthcare settings to avoid overcrowding in order to prevent SARS-CoV-2 transmission (2).

A systematic review commissioned by the World Health Organization attempted to analyse physical distancing measures in relation to coronavirus transmission (3). Physical distancing of <1 m (meter) was reported to result in a transmission risk of 12.8%, compared with 2.6% at distances ≥1 m, supporting physical distancing rules of 1 m or more (4). It is vital, therefore, to program access to facilities and services so that adequate physical distancing is guaranteed, and overcrowding is avoided, especially in the context of massive vaccination campaigns.

The problem of overcrowding in healthcare setting has mostly been faced in literature in the past with regards to Emergency Departments (5, 6). Literature regarding appointment scheduling in healthcare is vast (7–13). However, due to the inherent characteristics of medical services, it is difficult to predict exactly when a patient will arrive and how much time will be taken for the service (14). Even in the case of highly standardized practices such as vaccination, variability due to unpunctuality is always possible (15). This is especially true when targeting people during working hours, with some of them conducting busy and often unpredictable daily activities.

One of the approaches that could help solve this problem is “Queueing theory” (5, 6, 16). Queueing theory is a branch of applied mathematics that is used to predict the behavior of lines (also known as queues). A process is made up of a sequence of activities with various points of delay and stoppages. Flow units enter the process (arrivals), wait to be processed (queue), are processed (service), and then move to the next step in the process or exit it. The time spent in line or queue is a function of the configuration and discipline rate at which the flow units enter the system, the rate at which they are processed or served, the queue, and the population of flow units (5). To give some definitions: Server, s: the main unit providing service; Average Arrival Rate, λ: the average number of arrivals per unit of time; Average Service Rate, μ: the average number of units served or processed per unit of time. The Kendall notation is a well-established classification scheme. Three letters are used, M, G, and D, to designate the probability distributions of arrivals or service: M = Poisson distribution for rates or exponential distribution for times (the “M” stands for Markovian and/or “memoryless”); G = General/any distribution with a known mean and variance; D = Deterministic or constant.

“Queueing theory” has been mostly applied in healthcare (16) to enhance the performance of operating rooms (17), outpatients settings waiting times (8) and hospital bed management (18). It is starting to be applied to vaccination campaigns as well (2). A recent study of Safdar et al. (19) presents a novel application of DEA (Data Envelopment Analysis) for assessing the queuing process at an outpatients' department of a large public hospital in a developing country where appointment systems do not exist. DEA has been mostly applied to the efficiency comparison of hospitals (20–25). In this study the patient flow pathway considered consisted of two stages: consultation with a doctor and pharmacy. The DEA results indicated that waiting times and other related queuing variables included need considerable minimization at both stages. This is an important result of a study from a context in which appointment systems do not exist.

Our paper proposes the use of Queuing theory to provide a model for quantifying in advance how many healthcare workers to be booked daily for a vaccination site, taking into account the possible variations due to healthcare workers not being able to perfectly adhere to their scheduled arrival time, and to provide indicators of safety, with regards to physical distancing during the COVID-19 pandemic, and performance, with regards to utilization time of each unit and to the subjects' average waiting time.

## Methods

The main way to approach the subject of booking units to input in a healthcare service consists in appointment scheduling techniques. This is proved to be very efficient, however there is scarce literature and techniques to use in many real contexts such as ours in which reliance on subjects respecting their scheduled times would be unwise. In relation to this topic, scientific literature provides many tools such as Monte Carlo simulation (26), Discrete Event Simulation (DES) (27), agent-based simulation (28) and Data Envelopment Analysis (DEA) (19). We found queueing theory to be easy to be understood and applied by professionals from all healthcare sectors as it does not require high-demanding computing or technical skills, it is well supported by literature (8, 16–18) and it has already been successfully applied to vaccination settings (2).

A vaccination site for COVID-19 vaccination targeting healthcare workers based in a teaching hospital in Rome was studied. The Queuing theory model used is G/D/s/k/∞/FIFO. The characteristics of each parameter and the reason for making such choices regarding the type of queue model used are the following:

- G stands for a distribution with a known mean and variance. In order to derive a realistic coefficient of variation (CV) of arrival rate we therefore studied the recorded distribution of arriving subjects throughout the 3 days of major affluence, from 8th to 10th January 2021. These days were also the most homogenous with regards to number of subjects served and hours of activity (Table 1) and represented a situation of maximum stress for the system. Five-minute time slots for each of the three stations were available for booking. No more than 1 booking for time slot was possible;

- D this stands for service time. It was assumed to be constant (meaning a coefficient of variation equal to zero) and equal to 5 min, based on the average time needed for vaccination gained in our experience. This parameter was therefore used to determine the entity of each time slot;
- s stands for the number of stations, i.e., the total number of working stations. All vaccination sites were considered to have three stations, each one composed by: one public health medical doctor explaining the procedure, interviewing the healthcare worker and excluding any contraindication to the procedure (i.e., allergic to substances contained in the vaccine) and one nurse taking care of preparing and administering the vaccine shot;
- k stands for a maximum fixed length of the queue (or Lq). The maximum allowed length of the queue in all vaccination sites (determined in relation to the real-life setting waiting room space) was considered to be 20;
- ∞ stands for the entity of the population set. It was assumed to be infinite so as to give maximum possible variability to arrival rate throughout the simulations in order to find the most appropriate value for it;
- FIFO stands for the Queue discipline. The “first in–first out” order (“FIFO”, otherwise known as “FCFS” or first come–first served) was chosen, meaning that no subject could access the server before the end of the previous vaccination process; no system of priority other than arrival time was in fact considered;

The time unit was assumed to be 6 h for the first simulation, as this was the amount of time dedicated to vaccination in our original hospital-based setting. It was assumed to be 12 h for the second and third simulation, in order to have a double amount of time for vaccination but without making the staff exceed 12 consecutive working hours.

**Table 1:** Details of the activity regarding the three sampled vaccination days.

**Vaccination day**	**No. of subjects**	**Working hours (time slots)**	**Mean vaccinated per time slot (std. dev.)**	**Coefficient of variation (CV)**
8th January	174	6.66 (80)	2.18 (1.47)	0.67
9th January	179	6.25 (75)	2.39 (1.72)	0.72
10th January	174	6.00 (72)	2.41 (1.66)	0.69

Therefore, Queueing theory indicators to study the model were used.

For the safety analysis, to make sure adequate physical distancing was kept, we used average Length of queue (Lq);

For the performance analysis we used: ρ = percentage of the total time each station was in use, with 100 – ρ being a measure of resources underutilization; AWT = average subject's waiting time;

We therefore performed three simulations to study the activity using the aforementioned indicators in:

- a hypothetical setting modeled on the original;
- a hypothetical double-sized original setting;
- a hypothetical mass vaccination site.

The result of the simulations in each setting was confronted with a linear model booking system as the starting point and the value of the arrival rate was lowered unit by unit for each simulation.

For all graphs and analysis Microsoft Excel for Mac (Version 16.16.27-201012) was used. Example of formulas for Excel-based queueing model building are open access and freely available (for instance at https://www.csus.edu/indiv/b/blakeh/mgmt/documents/opm101supplc.pdf). As a G/D/k model tailored to the specific requirements of our study requires complex computational capacity, safety and performance indicators were obtained using an online calculator (29). The parameters of interest used in each simulation were defined in the Results section. Other parameters available were not modified from the default calculator mode and assigned as such: arrival batch size = 1; batch size (service process) = 1; coefficient of variation of service process (given service rate was a fixed parameter) = 0. The last 3 parameters, regarding failure behavior of the model, were assigned as: availability = 1; average down time = 0; coefficient of variation = 1.

The obtained values for queue length with decimal digits were rounded to the integer value. The obtained value for AWT was converted by multiplying it by the established time unit in minutes for each simulation.

**Figure 1:**
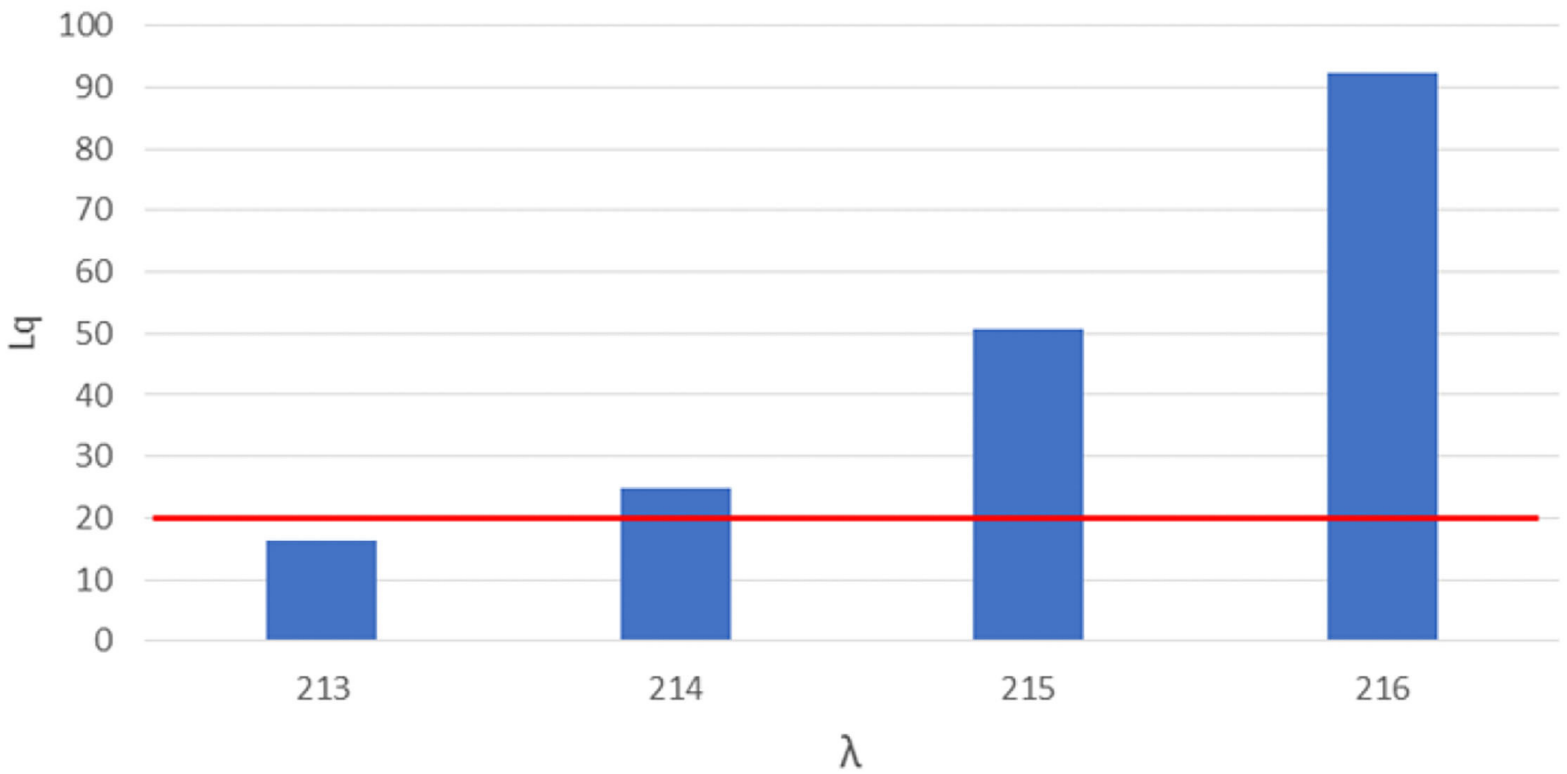
Number of queueing subjects (Lq) by one-unit increases in arrival rates (λ) in original setting. Maximum queue length evidenced in red line.

**Figure 2:**
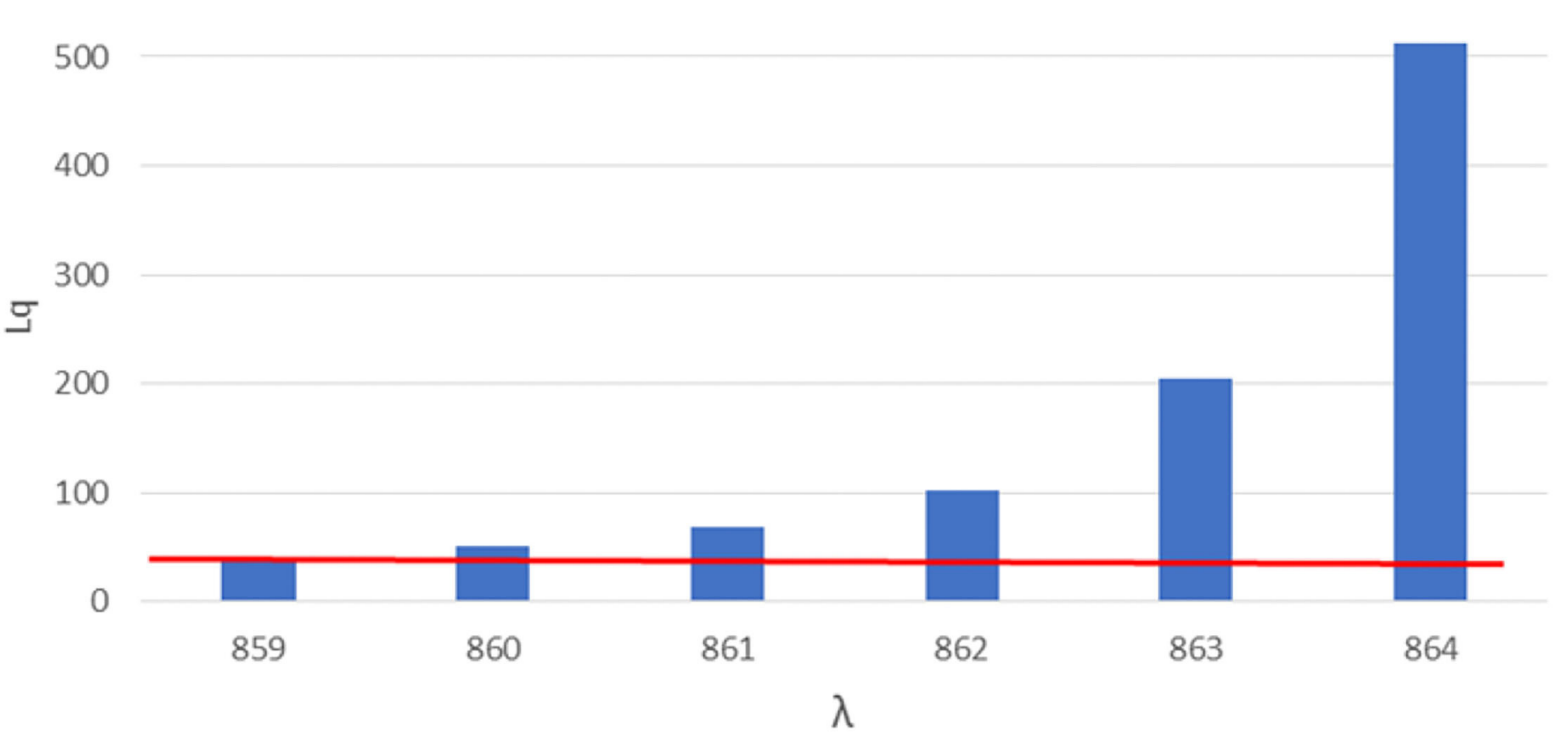
Number of queueing subjects (Lq) by one-unit increases in arrival rates (λ) in double-sized setting. Maximum queue length evidenced in red line.

**Figure 3:**
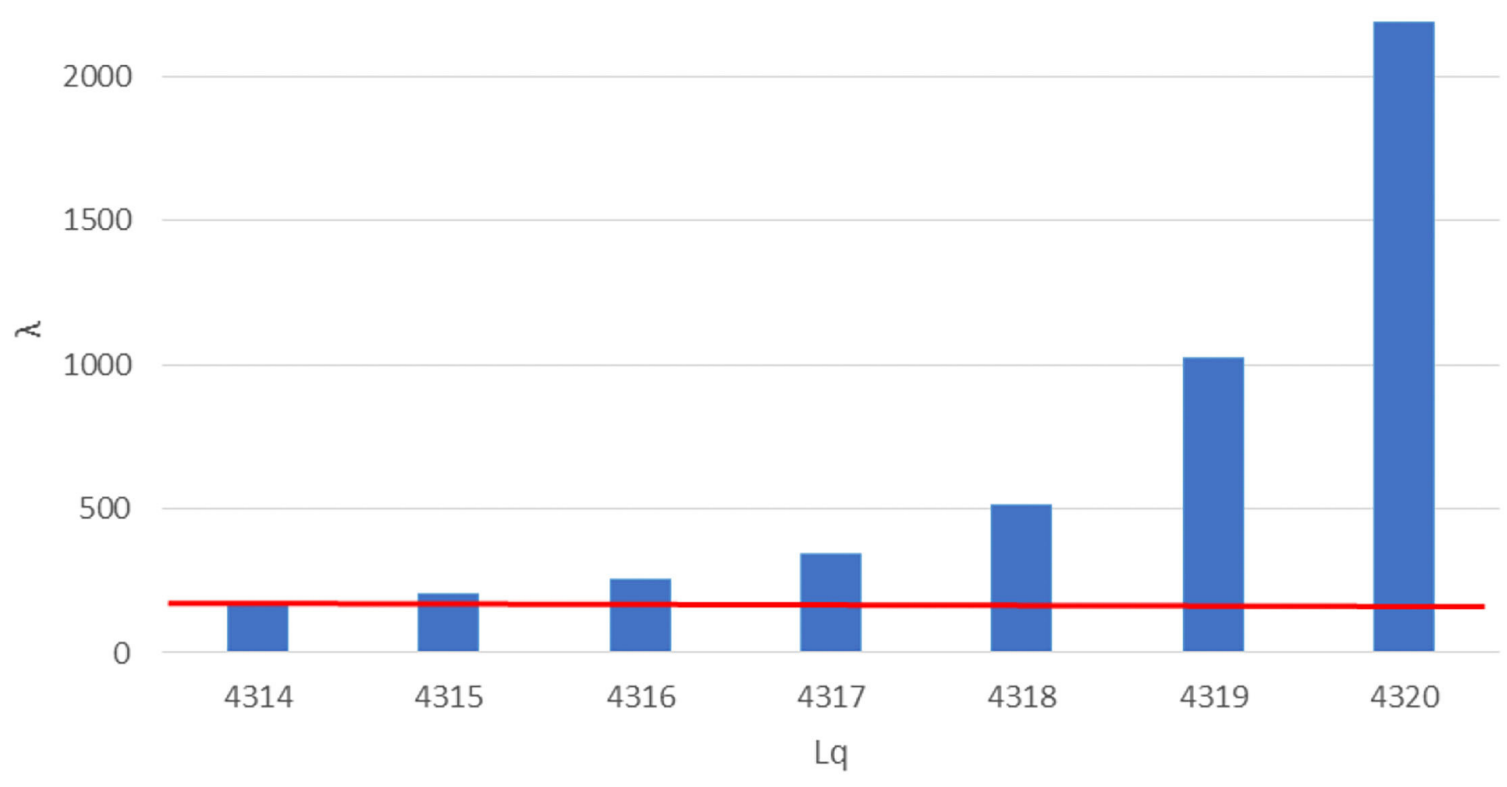
Number of queueing subjects (Lq) by one-unit increases in arrival rates (λ) in mass vaccination site. Maximum queue length evidenced in red line.

Figures 1–3 illustrate the following findings.

**Figure 4:**
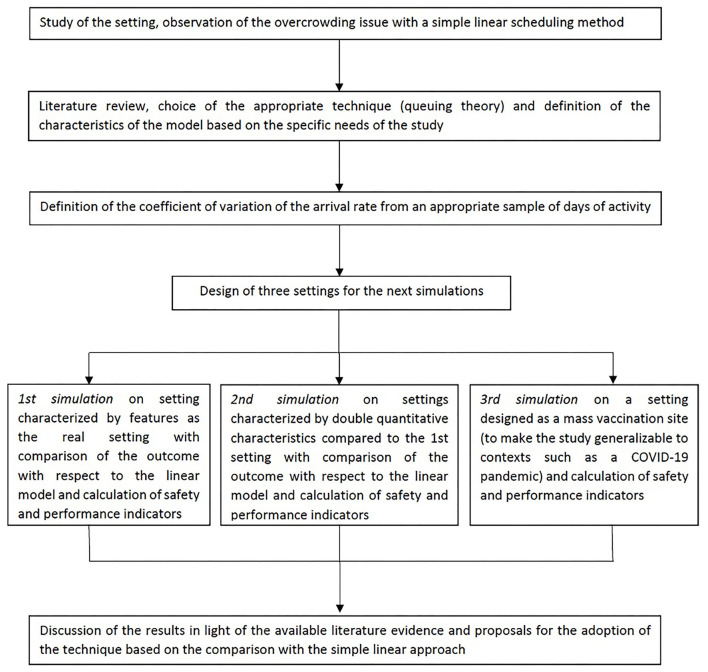
Flow chart illustrating the study process step-by-step.

Figure 4 shows a flow chart demonstrating how each step has been undertaken.

## Results

### Determining Arrival Distribution

Table 1 shows data regarding the activity during the three sampled vaccination days.

A mean CV of 0.69 across the 3 days was observed and therefore used for the simulations to determine arrival distribution. In fact, the percentage of time slots with more than 3 arriving subjects, which should amount to zero in an ideal setting with perfect adherence of subjects to the scheduling, were found to be, respectively, 10.90, 18.99 and 17.24% across the 3 days.

### Original Setting

For the first simulation we considered a service rate (the rate of served subjects per total working session) of 72 subjects served by each station, 3 stations (each station composed by a doctor-nurse unit) working in parallel, 5 min were designated as the time slot needed for each vaccination, the maximum number of subjects that could stay in the queue in compliance with the COVID-19 safety requirements in relation to the real-life space at disposal was 20, the total working time of the site was 6 h and 0.69 was the coefficient of variation of the arrival rate as determined in paragraph 3.1. A linear booking model was used as a starting point, multiplying the service rate by the number of working stations and obtaining 216 as the number of subjects (λ, arrival rate) to be booked for the day.

We therefore proceeded with a more complex approach based on Queueing theory, varying the arrival rate simulation by simulation starting with 216 and performing a safety and performance analysis on each simulation‘s outcome via the indicators mentioned in the Method section.

The safety analysis showed that, an arrival rate λ of 214, we would have 25 subjects in the queue (Lq), exceeding the safe queue limit by 5. We therefore performed another simulation and found 213 as the threshold arrival rate value for safety by which the queue amounts to 20 subjects.

The performance analysis showed that, with 213 as arrival rate we found 98.6% as the percentage of time each station was in use (ρ), which implies underuse of 1.4% of each station's time. 27.72 min were determined as the average time each subjects had to wait in line (AWT or average waiting time).

A difference of 3 subjects in determining the appropriate arrival rate was therefore found between a linear approach by simple multiplication (assuming a coefficient of variation of arrival rate equal to zero) and a more complex Queueing theory approach (assuming a coefficient of variation of arrival rate derived from a real-life setting observation).

However little could seem this difference, as shown in Figure 1, letting in a few more units could cause an exponential growth of Lq, with a difference of 3 more booked subjects (from 213 to 216) causing an increase of 112 subjects over the safe limit of the waiting queue (from 17 queueing subjects with an arrival rate of 213 to 128 queueing subjects with an arrival rate of 216).

Figure 1 shows the number of queueing subjects by one-unit increases in arrival rates in original setting, maximum queue length is evidenced in red line.

### Double-Sized Setting

For the second setting we considered a service rate (the rate of served subjects per total working session) of 144 subjects served by each station, 6 stations (each station composed by a doctor-nurse unit) working in parallel, 5 min were designated as the time slot needed for each vaccination, the maximum number of subjects that could stay in the queue in compliance with the COVID-19 safety requirements in relation to the real-life space at disposal was 40, the total working time of the site was 12 h and 0.69 was the coefficient of variation of the arrival rate as determined in paragraph 3.1. A linear booking model was used as a starting point, multiplying the service rate by the number of working stations and obtaining 864 as the number of subjects (λ, arrival rate) to be booked for the day.

We therefore proceeded with the approach based on Queueing theory, varying the arrival rate simulation by simulation starting with 864 and, as for the first setting, performing a safety and performance analysis on each simulation‘s outcome.

The safety analysis showed that, already at an arrival rate λ of 860, 51 subjects are found in the queue (Lq), exceeding the safe queue limit of 40 by 11 units. We therefore performed another simulation and found 859 as the safe arrival rate value by which the queue amounts to 40 subjects.

The performance analysis showed that, with 859 as arrival rate, we found 99.4% as the percentage of time each station was in use (ρ), which implies underuse of 0.6% of each station's time, and an average waiting time of 33.84 min.

A difference of 5 subjects in determining the appropriate arrival rate was therefore found between a linear approach by simple multiplication (assuming a coefficient of variation of arrival rate equal to zero) and a more complex Queueing theory approach (assuming a coefficient of variation of arrival rate derived from the observation a real-life setting).

However little could seem this difference, as shown in Figure 1, letting in a few more units could cause an exponential growth of Lq, with a difference of 5 more booked subjects (from 859 to 864) causing an increase of subjects over the safe limit of the waiting queue of 483 subjects (from 40 queueing subjects with an arrival rate of 859 to 523 queueing subjects with an arrival rate of 864).

Figure 2 shows the number of queueing subjects by one-unit increases in arrival rates in original setting, maximum queue length is evidenced in red line.

### Mass Vaccination Site

For the third setting we considered a service rate (the rate of served subjects per total working session) of 144 subjects served by each station, 30 stations (each station composed by a doctor-nurse unit) working in parallel, 5 min were designated as the time slot needed for each vaccination, the maximum number of subjects that could stay in the queue in compliance with the COVID-19 safety requirements in relation to the real-life space at disposal was 40, the total working time of the site was 12 h and 0.69 was the coefficient of variation of the arrival rate as determined in paragraph 3.1. A linear booking model was used as a starting point, multiplying the service rate by the number of working stations and obtaining 4,320 as the number of subjects (λ, arrival rate) to be booked for the day.

We therefore proceeded with the approach based on Queueing theory, varying the arrival rate simulation by simulation starting with 4,320 and, as for the first setting, performing a safety and performance analysis on each simulation‘s outcome.

The safety analysis showed that, already at an arrival rate λ of 4,315, 204 subjects are found in the queue (Lq), exceeding the safe queue limit of 40 by 4 units. We therefore performed another simulation and found 4,314 as the safe arrival rate value by which the queue amounts to 170 subjects.

The performance analysis showed that, with 4,314 as arrival rate, we found 99.8% as the percentage of time each station was in use (ρ), which implies underuse of 0.2% of each station's time, and an average waiting time of 33.84 min.

A difference of 6 subjects in determining the appropriate arrival rate was therefore found between a linear approach by simple multiplication (assuming a coefficient of variation of arrival rate equal to zero) and a more complex Queueing theory approach (assuming a coefficient of variation of arrival rate derived from the observation a real-life setting).

However little could seem this difference, as shown in Figure 1, letting in a few more units could cause an exponential growth of Lq, with a difference of 6 more booked subjects (from 4,314 to 4320) causing an increase of 750 subjects over the safe limit of the waiting queue (from 170 queueing subjects with an arrival rate of 4,314 to 855 queueing subjects with an arrival rate of 4,320).

Figure 3 shows the number of queueing subjects by one-unit increases in arrival rates in original setting, maximum queue length is evidenced in red line.

## Discussion

As anticipated in the Results section, the percentage of time slots with more than 3 arriving subjects, which should amount to 0.00% in an ideal setting with perfect adherence of subjects to the scheduling, were found to be, respectively, 10.90, 18.99 and 17.24% across the 3 days. This proves that unquestioned reliance on subjects respecting their scheduled times would be unwise.

With regards to safety analysis, comparing the results obtained by three simulations we can see that the number of subjects prevented from overcrowding the vaccination site increases exponentially with the increase in size and in the chosen parameters of the site.

With regards to performance analysis, we can see the highest performance in terms of percentage of stations' time in use and average waiting time is found in the mass vaccination site, followed by the double-sized setting and the original setting simulation.

We see how Queueing theory, allowing for performance and safety analysis, shows its greatest potential with mass vaccination sites, though proving useful in smaller setting as well.

We observed that the most effective model had, respectively, 5 and 10 times the number of servers, 5 and 10 times the number of places for queueing and twice and the same number of working hours of the second and first simulated models.

The opportunity of applying Queueing theory is supported by recent literature.

A study by Hanly et al. (2) makes an important use of this theory in modeling the entirety of vaccination sites. The experimenters conclude by reporting that “[..] queueing models can be used to simulate vaccination queues, estimate daily throughput based on given staff availability and inform service delivery”.

To conclude, our choice of the parameters in building different models for simulation was demonstrational. Future use of the model could therefore consist in studying, for instance, how many more subjects can be vaccinated, while ensuring maximum performance and safety of the site, by differentially increasing one parameter at a time to find the most suitable configuration in relation to the specific organizational requirements of the user.

### Study Limitations

This study has some limitations.

First, even though a distribution of variability of service rate could have proven useful in improve adherence to real-life setting (some subjects take more or <5 min to be vaccinated according, for instance, to the complexity of the health status assessment), such data were unfortunately not recorded. Service rate CV was therefore assumed equal to zero for the sake of our simulations. A “D” (deterministic) instead of a “G” distribution was adopted. Based on our experience, however, we reckon that much of the variability in the process is attributable to arrival rate rather than service rate. We nonetheless acknowledge this limitation and propose to account for it in future studies.

Second, many vaccination sites include a second waiting room for a brief (15 min) medical observation of vaccinated subjects. This element could be implemented into the system, as well as, for instance, considering doctor's room and nurses' room as separate units and studying queues forming up to each of them, with the use conjoint probability. We refer to the topic of Markovian network processes (30) for further study on this matter.

Third, there is plenty of literature that implements Queueing theory and other techniques to reduce appointment scheduling variability (7–13). However, these techniques have not yet been diffusely adopted in common vaccination practice worldwide and, therefore, our use of Queueing theory directly on determination of a proper arrival rate might prove all the more useful or, at least, represent a starting point for new techniques' implementation. As anticipated, Hanly et al. make ample use of it in modeling the entirety of vaccination sites (2). We furthermore stress the utility of this technique in giving specific indicators by which both safety and performance analysis can be performed on vaccination sites already in use, without the need for recording every activity's parameter, giving thus the chance for optimization. For instance, without the need for recording our waiting queue length throughout the activity, we retrospectively acknowledge that we might have at times exceeded maximum safety length, while also being able to quantify a potential resources underuse of 1.4% with only the electronically recorded distribution of arrival rate as a starting point.

## Conclusions

“Queueing theory” has been mostly applied in healthcare (18) to enhance the performance of operating rooms (16), outpatients settings waiting times (9) and hospital bed management (31). It is starting to be applied to vaccination campaigns as well (2, 32).

We propose the adoption of this tool by public health figures involved in vaccination practices as it enables to quantify ahead of time, using specific indicators, the outcome of organizational choices on both safety and performance of vaccination sites.

This tool can be used to develop the policy framework for improving the operations throughout healthcare and vaccination programs by catering to the requirements of specific stakeholders such as managers, professionals, and patients. Its application would in fact increase the efficiency of healthcare operations enabling managers to control costs but also adding value for both healthcare workers, who perceive the better management of their working time and modalities, and patients, who perceive the better quality of the service provided. Promoting a tool such as this one could help contribute to some of the strategies indicated by the Global Alliance for vaccines and Immunization (GAVI), as reformulated by Kamara et al. (33), such as vaccine logistics and stock management and training, improving service delivery and reducing vaccine wastage.

Further work applying Queueing theory model in vaccination as well as other healthcare settings, considering the urgency deriving from the current pandemic, is much needed. For instance, more complex evaluations connecting the outcome of organizational choices informed by queueing theory-based models on human and economic resources would be of great interest and use for the scientific literature.

## Data Availability Statement

The raw data supporting the conclusions of this article will be made available by the authors, without undue reservation.

## Author Contributions

MD: conceptualization, methodology, visualization, software, and formal analysis. MD, AI, GM, AD, and RG: data curation, investigation, and writing—original draft preparation. MD and DP: validation. PL, GD, and WR: resources, supervision, and project administration. MD, GD, PL, and DP: writing—review and editing. All authors contributed to the article and approved the submitted version.

## Conflict of Interest

The authors declare that the research was conducted in the absence of any commercial or financial relationships that could be construed as a potential conflict of interest.

## Publisher's Note

All claims expressed in this article are solely those of the authors and do not necessarily represent those of their affiliated organizations, or those of the publisher, the editors and the reviewers. Any product that may be evaluated in this article, or claim that may be made by its manufacturer, is not guaranteed or endorsed by the publisher.
